# Impact of delipidated estrous sheep serum supplementation on *in vitro* maturation, cryotolerance and endoplasmic reticulum stress gene expression of sheep oocytes

**DOI:** 10.1371/journal.pone.0198742

**Published:** 2018-06-18

**Authors:** Natalibeth Barrera, Pedro C. dos Santos Neto, Federico Cuadro, Diego Bosolasco, Ana P. Mulet, Martina Crispo, Alejo Menchaca

**Affiliations:** 1 Instituto de Reproducción Animal Uruguay, Fundación IRAUy, Montevideo, Uruguay; 2 Unidad de Animales Transgénicos y de Experimentación, Institut Pasteur de Montevideo, Montevideo, Uruguay; Peking University Third Hospital, CHINA

## Abstract

High lipid content of oocytes and embryos in domestic animals is one of the well-known factors associated with poor cryosurvival. Herein, we wanted to determine whether the use of delipidated estrous sheep serum during *in vitro* maturation (IVM) of ovine oocytes reduces the cytoplasmic lipid droplets content and improves embryo development and cryotolerance after vitrification. Cumulus oocytes complexes (COCs) were matured *in vitro* for 24 h in medium supplemented with whole or delipidated estrous sheep serum prior to vitrification. Neutral lipid present in lipid droplets of COCs, cleavage rate, embryo development rate on Day 6 and Day 8, and hatching rate on Day 8, were compared among experimental groups. Endoplasmic reticulum stress genes were evaluated in *in vitro* matured COCs under different lipid conditions prior to vitrification. The lipid droplets’ content (mean fluorescence intensity) of oocytes cultured with IVM media supplemented with delipidated serum was lower than COCs matured with whole serum (7.6 ± 1.7 vs. 22.8 ± 5.0 arbitrary units, respectively; P< 0.05). Despite IVM treatment, oocytes subjected to vitrification showed impaired competence compared with the non-vitrified groups (P<0.05). No significant differences in embryo production were observed in non-vitrified COCs after maturation in delipidated or whole serum (33.4±4.9 vs 31.9 ±4.2). COCs matured in delipidated serum and subjected to vitrification showed increased expression of *ATF4*, *ATF6*, *GRP78*, and *CHOP10* genes (ER stress markers). Collectively, our results demonstrate that although supplementation of IVM medium with delipidated estrous sheep serum reduces the presence of cytoplasmic lipid droplets in oocytes after maturation, oocyte cryotolerance is not improved. Notably, the expression of genes associated with the unfolded protein response (UPR) was increased in COCs, with fewer lipid droplets subjected to vitrification, suggesting that oocyte cryopreservation is associated with ER stress and activation of adaptive responses.

## Introduction

Within the last decade, there have been significant advances in methods to improve oocyte cryotolerance. Difficulties associated to oocyte cryopreservation are related with inherent structural and physiological features. Particularly in domestic animals, the high intracellular lipid content of oocytes adds greater complexity to cryopreservation. Different strategies have been developed to reduce lipid droplets in oocytes including mechanical removal and pharmacological options[1–3]. Furthermore, it is generally accepted that *in vitro* embryo production (IVEP) systems are not as efficient as *in vivo* embryo production, mainly due to lower oocyte competence acquisition when maturation is induced under *in vitro* conditions [1,4,5]. Although the underlying mechanisms behind oocyte competence have not yet been fully elucidated, there is increasing evidence that oocyte metabolism and the somatic environment play crucial roles in determining oocyte growth and developmental competence [6]. Lipid metabolism provides a good source of energy during oocyte maturation upon demand. For example, fatty acids stored within lipid droplets provide adenosine triphosphate (ATP) molecules through β-oxidation, largely serving as an energy source during oocyte maturation and early embryo development [7]. Therefore, components within the culture medium supplied to the cumulus-oocytes complexes (COCs) during *in vitro* maturation have the potential of affecting oocyte competence [8].

Studies show that when oocytes are matured *in vitro*, energy substrates as fatty acids provided through culture media can lead to increased intracellular lipid droplet (LD) accumulation [9–11] and alter oocyte metabolism, thereby affecting their quality. A recent report showed that exposure of mice COCs to high lipid content follicular fluid was associated with endoplasmic reticulum (ER) stress induction and led to a decrease in oocyte competence [12]. Moreover, increased accumulation of lipids in oocytes was correlated with reduced cryopreservation resistance [13]. It is well established that in sheep and other species, *in vitro* generated embryos exhibit reduced cryotolerance [14,15]. The process of cryopreservation leads to multiple changes, including structural modifications and alterations in gene expression patterns [16]. For example, increased expression of genes associated with ER stress has been reported after oocyte cryopreservation. In domestic animals, the high lipid content in oocytes may pose challenges for cryopreservation [10]. Specifically, bovine, ovine, and porcine oocytes are highly susceptible to cryoinjuries, with the majority of studies reporting a blastocyst rate of 0 to 20% [2,17–20] after oocyte vitrification. Additional studies have suggested that tolerance of oocytes to chilling injuries can be increased when cytoplasmic lipid content is reduced, thereby improving cleavage and blastocyst rates[10,21]. Interestingly, the introduction of controlled stress during *in vitro* culture led to improved cleavage and blastocyst rates and suggests that oocytes perform better under specific types of stress signals [22,23]. Induction of pathways related to ER stress have been reported in oocytes subjected to vitrification [12]. ER stress is a mechanism associated with excess intracellular lipid accumulation in COCs [24]

The addition of animal serum to IVEP medium is a standard practice and helps promote oocyte maturation and subsequent embryo development [25]. During culture of ovine oocytes, estrous serum is routinely used to supplement the maturation medium, since it is known that it contains a range of beneficial components, including hormones, growth factors, amino acids, binding proteins. Conversely, estrous serum also provides a significant source of lipids. Therefore, the reduction of lipid content is necessary to improve *in vitro* embryo cryosurvival and blastocyst rates in IVP systems. One strategy to reduce lipid exposure involves incorporation of serum-free media during IVM. [26]. Nutrient restriction in the maturation medium promotes the use of the oocyte’s own endogenous reserves, thereby reducing the amount of intracellular lipids [10]. However, oocyte competence is impaired under serum-free culture conditions compared to undefined media in which serum has been added [21]. Alternatively, restriction of lipid content in the serum may be a more optimal strategy to reduce lipid droplets in the oocyte and may improve oocyte cryosurvival.

The objective of this study was to determine whether supplementation of IVM medium with delipidated estrous sheep serum affects the amount of cytoplasmic LD in *in vitro* matured sheep oocytes. Furthermore, we investigate whether intracellular lipid content variations in oocytes is associated with oocyte developmental competencies, cryotolerance, embryo production, and ER stress.

## Materials and methods

### Experimental design

**Fig 1:**
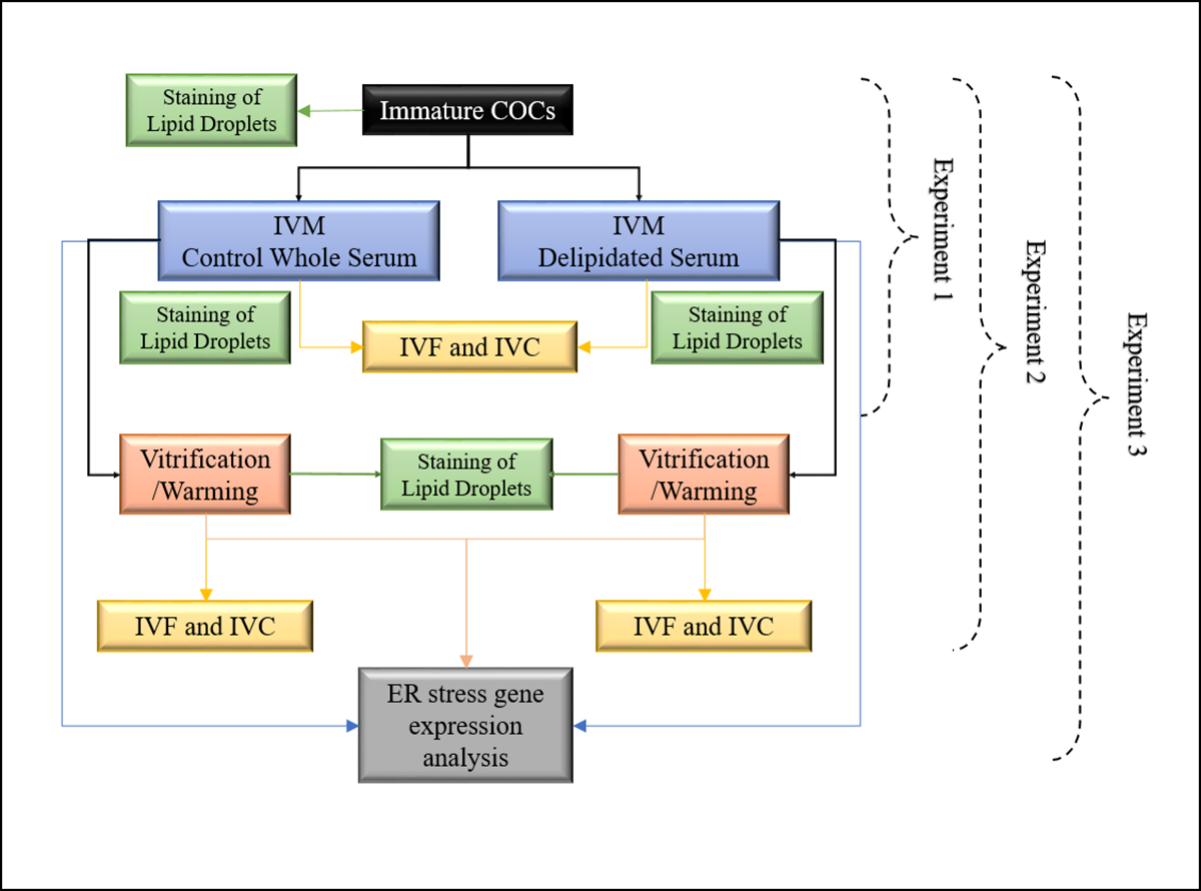
Experimental design. Schematic representation for determination of the effect of control whole estrous sheep serum vs. delipidated serum used during in vitro maturation of cumulus oocytes complexes (COCs) on oocyte lipid content and: embryo development (Experiment 1), cryotolerance after vitrification (Experiment 2), and expression of endoplasmic reticulum (ER) stress genes (Experiment 3).

Three experiments were conducted by using a total of 2,986 COCs at Fundación IRAUy and Transgenic and Experimental Animal Unit of Institut Pasteur of Montevideo, Uruguay. The experimental design is shown in Fig 1. Unless stated otherwise, all media and chemicals were purchased from Sigma (St. Louis, MO, USA).

#### Experiment 1

Experiment 1 was conducted to evaluate the effect of the lipid content (based on the concentrations of Triglycerides, total Cholesterol, and non-esterified fatty acid (NEFAs)) of IVM medium supplemented with estrous sheep serum on: (a) neutral lipid stored in LD of *in vitro* matured oocytes, and (b) oocyte developmental competence. A total of 866 COCs were collected from slaughterhouse ovaries and subjected to IVM in a supplemented medium with whole estrous sheep serum (Control whole serum group, n = 452) or delipidated estrous sheep serum (Delipidated serum group, n = 414). Neutral lipid present in LD of partially denuded COCs, cleavage rate, development rate on Day 6 and Day 8, and hatching rate on Day 8, were compared between the two experimental groups (Fig 1). Seven replicates of this experiment were performed for each treatment group.

#### Experiment 2

Experiment 2 was conducted in order to further investigate the effect of vitrification on the survival rates of oocytes previously matured under different lipid content conditions. Viable immature COCs (1280) were randomly assigned to two experimental groups consisting of IVM medium supplemented with control or delipidated estrous serum sheep.

After IVM, COCs were submitted to IVF and IVC (Control whole serum group, n = 344; Delipidated serum group, n = 357); or were vitrified using the Cryotop method (Control whole serum + vitrification group, n = 288; Delipidated serum + vitrification group, n = 291) before IVF and IVC. Oocyte lipid content was assessed by LD staining before and after vitrification/warming, and cleavage rate, development rate on Day 6 and Day 8, and hatching rate on Day 8 were compared among groups (Fig 1). A total of thirteen replicates of this experiment were performed for each treatment group.

#### Experiment 3

Experiment 3 was performed in order to determine whether *in vitro* matured COCs under different lipid conditions followed by vitrification expresses different levels of ER stress markers. A total of 840 COCs were used to determine ER stress gene expression (*ATF4*, *ATF6*, *GRP78*, and *CHOP10*) by real time PCR in five experimental groups (Immature, Control whole serum group, Delipidated serum group, Control whole serum + Vitrification group, and Delipidated serum + Vitrification group). Seven replicates of this experiment were performed using 30 COCs per replicate for each treatment group.

### Estrous sheep serum source, lipid removal and lipid determinations

For serum preparation, blood samples were collected from 15 ewes in estrus previously treated with a hormonal protocol for estrous synchronization [27]. The procedure was approved by the Internal Animal Care Committee of Fundación IRAUy that is certified by the National Council of Animal Care of Uruguay. The blood was allowed to clot at room temperature during one hour and then centrifuged at 1500 g for 20 min at 4°C. Serum was collected, pooled and heat inactivated at 56°C for 30 min. Lipid removal from serum was performed by using Cleanascite™ (Biotech Support Group, NJ, USA) according to the instructions provided by the manufacturer. In brief, 1 ml of Cleanascite^TM^ was added to 4 ml of serum (1:4 v/v), samples were gently mixed for 10 min at room temperature, lipid’s agglomeration was improved by incubation at 4°C for 1 h, samples were centrifuged at 1000 g for 15 min at 4°C, and then, the supernatants were pooled and filtered with a 0.22 μm filter.

Aliquots from the whole and the delipidated estrous sheep serum (five samples of each group, same batch) were analyzed for Triglycerides, total Cholesterol, and NEFAs by using the commercial kits TG color GPO/PAP AA, Colestat enzimático (Wiener Lab, Rosario, Argentina), and NEFA-HR (2) (Wako Chemicals USA, Inc., Richmond, VA, USA), respectively. All enzymatic colorimetric assays were performed according to the manufacturer's instructions. Measurements were obtained with a biochemical analyzer (Vitalab Selectra-2 Merck, Darmstadt, Germany). The lipid removal efficiency was 54.4% for total cholesterol, 21.2% for triglycerides and 30.6% for NEFAs. For this reason, delipidated serum was in fact partially delipidated. Data presented on Table 1 show the mean values and inter-assay coefficient of variation (CV) for each metabolite.

**Table 1:** Lipid content of whole and delipidated estrous sheep serum (prior to be added to maturation medium).

	Control whole serum (mmol/L)	CV (%)	Delipidated serum (mmol/L)	CV (%)
Triglyceride	0.283 ± 0.01	5.150	0.223 ± 0.001	11.2236
Total cholesterol	1.80 ± 0.00	0.000	0.820 ± 0.007	6.061
Non-esterified fatty acid	0.718 ± 0.003	7.223	0.498 ± 0.017	3.433

Values are Means ± SD of 5 measurements of the same serum batch.

### Oocyte collection

Sheep ovaries were collected from the slaughterhouse and transported to the laboratory within 1 h in saline solution with 50 IU/ml of Penicillin and 50 μg/ml of Streptomycin at 35–37°C. The COCs were aspirated from antral follicles (2 to 6 mm) using a 21 gauge needle and a 5 ml syringe containing 0.5 ml of collection medium containing HEPES-buffered Tissue Cultured Media 199 (TCM 199) supplemented with 5 IU/ml of Heparin, 50 IU/ml of Penicillin, 50 μg/ml of Streptomycin, and 0.3% fatty acid-free Bovine Serum Albumin (BSA). Only COCs surrounded with three or more layers of granulosa cells and with homogeneous cytoplasm were selected for maturation purposes.

### *In vitro* maturation (IVM)

Embryo production was performed according to the standard operative procedures of our laboratory using the method described by Menchaca et al. (2016) [3]. Briefly, selected COCs were washed three times in a washing medium containing TCM 199 + HEPES supplemented with 50 IU/ml of Penicillin, 50 μg/ml of Streptomycin, and 0.3% fatty acid-free BSA. Groups of 25–30 COCs were placed into 100 μl droplets of maturation medium under mineral oil at 39°C in a humidified atmosphere of 5% CO_2_ in air for 22–24 hours. For IVM, the medium was supplemented with either 10% estrous sheep serum (whole or delipidated), 10 μg/ml FSH, 10 μg/ml LH, 100 μM Cysteamine, 50 IU/ml Penicillin, and 50 μg/ml of Streptomycin.

### *In vitro* fertilization (IVF)

COCs were removed from maturation drops and washed three times in IVF medium consisting of synthetic oviduct fluid (SOF), 2% estrous sheep serum, 10 μg/ml Heparin, and 10 μg/ml Hypotaurine. For fertilization (Day 0), frozen semen from a single ram previously frozen and tested in our lab for IVF was used. Motile spermatozoa were obtained by swim-up method [28] with slight modifications. COCs were placed into 100 μl of IVF medium, covered with mineral oil and inseminated with 1 x 10^6^ spermatozoa/drop. *In vitro* fertilization was carried out at 39°C in 5% CO_2_ with humidified atmosphere for 22 hours.

### *In vitro* culture (IVC)

Presumptive zygotes were denuded by gentle pipetting and were washed three times in drops of culture medium (SOFaaBSA bicarbonate buffered) containing 5% (v/v) Basal Medium Eagle (BME)-essential amino acids, 2.5% (v/v) Minimum Essential Medium (MEM)-nonessential amino acids, and 4 mg/ml of BSA. Embryonic development took place in a humidified atmosphere of 5% CO_2_, 5% O_2_, 90% N_2_ at 39°C and the medium was renewed on Day 3 and Day 6 [3]. The percentage of cleaved embryos on Day 2 was recorded (2–8 cell embryos/total oocytes). Development rate on Day 6 (number of morulae and blastocysts) and on Day 8 (number of blastocysts) were expressed on the basis of number of presumptive zygotes at the onset of IVC. Percentage of hatching blastocysts on Day 8 (hatching rate) was determined on the basis of the total number of blastocysts on the same day.

### Oocyte-vitrification and warming procedure

For the experimental groups submitted to vitrification (Experiment 2 and 3), cryopreservation was performed using the Cryotop method first described by Kuwayama et al. (2005)[29]. This method was performed using methodology and media previously reported by our group [14,15]. Following IVM, COCs were mechanically denuded by exposure to 0.1 mg/ml of hyaluronidase at 37°C for 30 seconds through pipetting using a 200 μl pipette tip. Partially denuded COCs were washed three times in a washing medium. Oocytes were first equilibrated at room temperature for 15 min in TCM 199 medium supplemented with 20% Fetal Bovine Serum (FBS), Basic Solution (BS) containing 7.5% (v/v) Ethylene Glycol (EG) and 7.5% (v/v) Dimethyl sulfoxide (DMSO), referred to as Equilibrium Solution (ES). Four oocytes were equilibrated at the same time. They were checked for recovery of the initial shape before the vitrification step. Following equilibration, oocytes were placed in a Vitrification Solution (VS) containing BS supplemented with 15% (v/v) EG, 15% (v/v) DMSO, and 0.5 M Sucrose. After 90 s in this solution, oocytes were placed on the Cryotop device (Kitazato Biopharma, Fujinomiya, Japan) in a minimum volume (e.g. <0.1 μl) and immediately submerged in liquid nitrogen. No more than four oocytes were loaded per Cryotop device. For warming, the Cryotop was removed from the liquid nitrogen and instantly placed in a solution containing BS plus 1.0 M sucrose at 37°C. After 1 minute, the oocytes were transferred to a solution consisting of BS plus 0.5 M Sucrose for 3 minutes at RT. Finally, a 5minute wash followed by a 1 minute wash was performed with BS at RT. The oocytes were then placed in IVM medium at 39°C with 5% CO_2_ humidified atmosphere for 2 h before IVF to allow microtubule repolymerization [30].

### Lipid droplet staining

In Experiment 1 and 2, BODIPY 493/503 dye (Invitrogen, Carlsbad, CA, USA), which stains intracellular neutral lipids, was used to localize LD. The neutral lipid dye BODIPY493/503 has been used to demonstrate differences in oocyte lipid content on a variety of species including mice, cows, sheep, pigs, and humans [8]. The methodology previously described for bovine oocytes was followed with few modifications [31]. COCs were partially denuded by exposure to 0.1 mg/ml of Hyaluronidase at 37°C for 30 s through pipetting using a 200 μl tip. Oocytes were washed three times in serum-free Polyvinylpyrrolidone in Phosphate-buffered saline (PBS-PVP; 0.2% w/v). COCs were fixed in 4% paraformaldehyde at 37°C for 1 h and washed twice in PBS-PVP. Oocytes were allowed to permeate for 30 min in PBS containing 0.1% (w/v) Saponin, 0.1 M Glycine (PBS-S). The DNA was stained with 10 μg/ml of TO-PRO-3 (Molecular Probes, Eugene, OR) for 20 min and subsequently washed in PBS-S. Next, LDs were stained with BODIPY 493/503 in PBS (20μg/ml) for 1 h in the dark, and oocytes were washed three times in PBS-PVP. Oocytes were then placed on a glass slide covered with 80% glycerol (in PBS) and sealed with a microscope slide.

### Lipid droplet determinations

Lipid droplet content was determined in Experiment 1 and 2. Images of oocytes were obtained using a confocal laser scanning microscope (Model LSM 800; Zeiss, Thornwood, NY, USA) attached to an inverted microscope (Model AxioObserver Z1; Zeiss, Thornwood, NY, USA) at 25X magnification. BODIPY 493/503 and TO-PRO-3 were subsequently excited with diode 488 nm lasers and diode 640nm laser. Emitted light was selected with emission detection wavelengths ranges for BODIPY 410–617 nm and for TOPRO-3 645–700 nm. Images were reconstructed using ZEN 2.1 software (Blue edition). From the *in vitro* matured groups only metaphase II stage oocytes were analyzed for lipid determination. Using ImageJ v.1.44g software, sum slices Z-projection was generated to a stack of images, same number of slices was used for each projection created (from 1 to 30 endpoint slice). The BODIPY fluorescence (arbitrary units of fluorescence) in the oocyte was determined after selection of the area covering entire ooplasm and the background region of each partially denuded oocyte [4]. After background correction Integrated density (Int Den) was calculated using Image J v.144- software. Finally IntDen mean ± SEM for each experimental group was determined.

### RNA isolation and reverse transcription

For RNA isolation, 30–35 COCs from each group of Experiment 3 (Immature, Control whole serum group, Delipidated serum group, Control whole serum + vitrification group, and Delipidated serum + vitrification group) were denuded, washed in PBS, and placed into a 1.5 ml microcentrifuge tube. The tubes were immediately submerged in liquid nitrogen and stored at -80°C until RNA extraction. Total RNA was isolated using the RNeasy Plus Micro Kit (QIAGEN, Hilden, Germany) according to the manufacturer’s instructions. The extracted RNA concentration and purity was estimated using a ND-1000 spectrophotometer (NanoDrop Technologies, Delaware USA). Sample purity was assessed using the A260/ A280nm ratio with expected values between 1.8 and 2.0. Reverse transcription was carried out with 50ng of total RNA using SuperScript®III First-Strand Synthesis System for RT-PCR (Invitrogen^™^) and a random hexamer primer in a final reaction volume of 10 μl according to the manufacturer’s instructions. The cDNA synthesis reactions were carried out at 25°C for 5 min for annealing, 50°C for 50 min for extension, followed by enzyme heat inactivation at 85°C for 5 min.

### Real-time polymerase chain reaction

For Experiment 3, gene expression was assessed by quantitative real-time PCR (qPCR). Oligonucleotide primers were designed for *GRP78*, *CHOP10*, *ATF4*, and *ATF6* using NCBI Primer Blast. Genes were selected as markers of activation of UPR, signaling branch activated under ER stress[32]. The primer sequences for *PPIA* and *TUBB* were taken from a published report where those genes were found most stable for normalization when random hexamers were used for cDNA priming in ovine oocytes [33]. Expected fragment size and GenBank accession numbers are listed on Table 2. All primers were synthesized by IDT (Integrated DNA Technologies, Coralville, IA, USA). The PCR mix in each include 5 μl of Power SYBR Green PCR Mix (2X) (Applied Biosystems, UK), 2μl of nuclease-free water, 1 μl of each forward and reverse primer pair (10 μM), and 1 μl of cDNA in a final volume of 10 μl. The PCR was carried out on an Eco™ Real-time PCR System (Illumina, San Diego, USA). The program used for the amplification of the genes consisted of an enzyme activation step of 10 min at 95°C followed by 45 cycles of PCR of a 15s denaturation at 95 ˚C, 60 s annealing/extension at 60 ˚C and a dissociation step consisting of 95°C for 15 s, 60°C for 15 s and finally 95°C for 15 s. At the end of the PCR reactions, melt curve analyses were performed for all genes to confirm the integrity of PCR products and specificity by the presence of a single peak. All samples were run in duplicate and mean value was used for calculations. Standard curves were created for each gene using a 3-fold dilution of cDNA and used to calculate individual real time PCR efficiencies (E) according to the formula %E = (10^−1/slope^– 1) × 100 [34]. The data generated by Eco Real-Time PCR System Software v5.0 (IIlumina, CA, USA) were transferred to Microsoft Excel for analysis.

**Table 2:** Details of oligonucleotides used for qPCR.

Gene symbol	Gene name	Primer sequence	Product Size (bp)	Accession number
*PPIA*	Peptidylprolyl isomerasa A	F: ATTTATGTGCCAGGGTGGTG	158	AY251270
		R: ACTGGGAACCATTTGTGTTGG		
*TUBB*	Beta-tubulin	F: CAGAGCAAGAACAGCAGCTACTT	228	AF035420
		R: GTGAACTCCATCTCGTCCATGCCCTC		
*ATF4*	Activating Transcription Factor 4	F: CGAGGTGTTTGTGGGGGACT	149	GAAI01000637.1
		R: AGGAGCCTGCCTTAGCCTTG		
*ATF6*	Activating Transcription Factor 6	F: GCTCTCTCAGCCTACCGTGG				130	AY942654.1
		R: CACAGGGGCTGGTACCACAT					
*GRP78 (HSPA5)*	Glucose regulated protein-78	F: AGCCCTATAGCTGCCTGCTG				131	DQ029323.1
		R: CCACGTCCTCCTTCTTGTCC					
*CHOP10 (DDIT3)*	C/EBP homologous protein	F: TGGAAGCCTGGTATGAGGAC				126	AY943948.1
		R: GAGAGGCAGGGTCAAGAGTG					

F, forward primer; R, reverser primer

Product size in base pairs.

All target gene transcriptions were expressed as an n-fold difference relative to the calibrator (Control serum group). Different amplification efficiencies for individual genes were considered [35]. The geometrical mean of two internal reference genes (*TUBB* and *PPIA*) was used to correct the raw values for the genes of interest.

### Statistical analysis

Statistical analysis was performed using Infostat software (Cordoba, Argentina). The criterion of data normality was evaluated by the Shapiro-Wilk test and percentage data was subjected to arcsine transformation and expressed as Mean ± SEM. Significance differences in Experiment 1 were tested by a two-way analysis of variance (ANOVA) followed by Tukey’s test when variables were normally distributed. A non-parametric Kruskal-Wallis test was conducted for comparison of variables that did not follow normal distribution. For Experiment 2 and 3, in which 2x2 factorial design was performed, mixed models were used with fixed effect for cryopreservation (vitrification or not), serum (whole or delipidated) and its interaction, and the replicate as random effect (13 replicates for Experiment 2 and seven replicates for Experiment 3). Values of p less than 0.05 were considered statistically significant.

## Results

### Experiment 1

**Fig 2:**
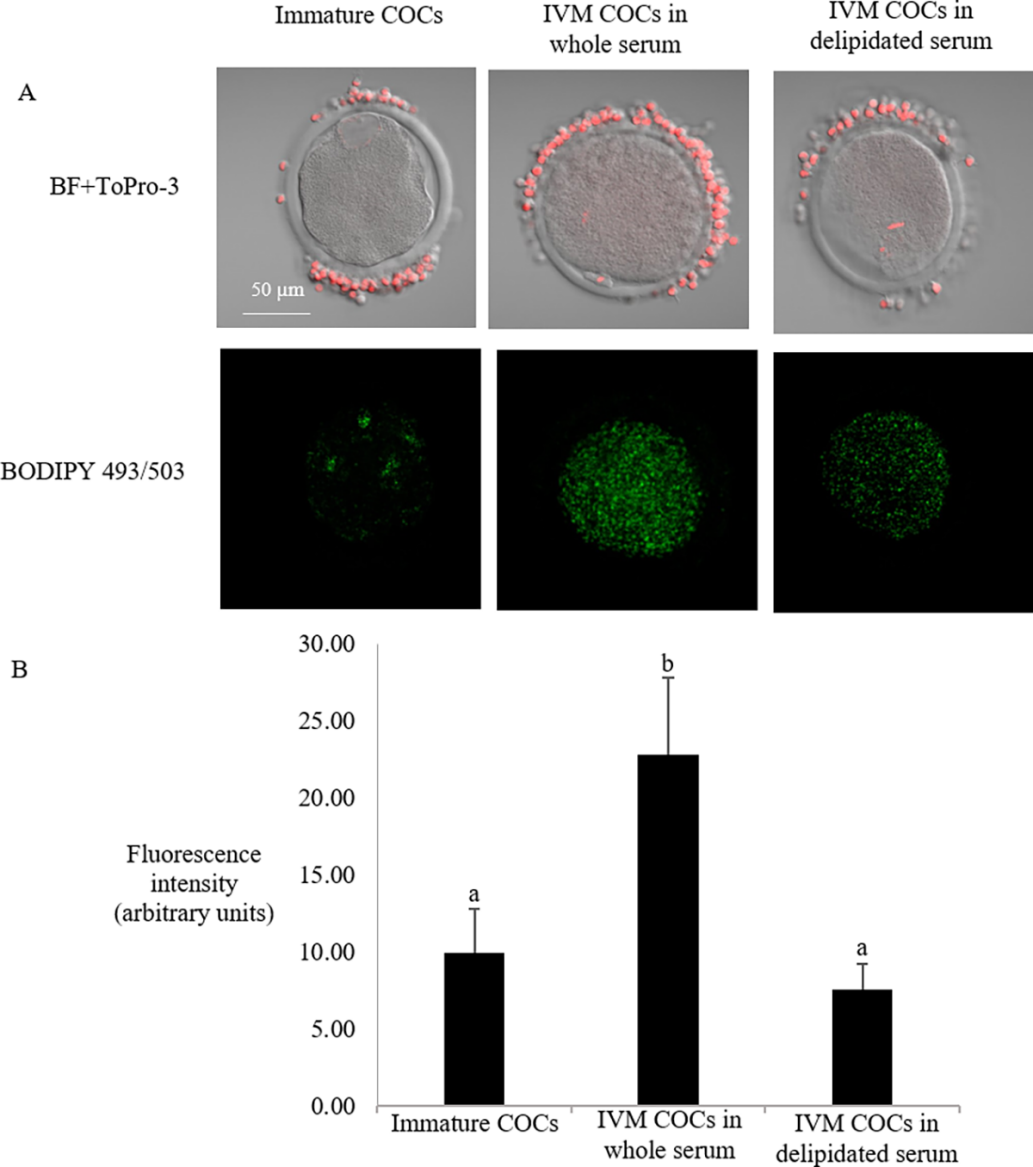
Lipid quantification of partially denuded COCs *in vitro* matured with control whole serum or delipidated serum (Experiment 1). a) Nuclei of cells were stained with ToPro-3 (red) and merged with bright field image (BF). Neutral lipid staining with BODIPY 493/503 (green) show an increase in lipid droplets of oocytes after exposure to IVM media supplemented with control whole serum. b) Comparison of the lipid content of immature (Immature, n = 23) and COCs in vitro matured in a medium supplemented with control whole (n = 24) or delipidated (n = 24) estrous sheep serum. Values are expressed as average of BODIPY fluorescence intensity in the ooplasma per area ± SEM. a vs b indicates significant differences (P<0.05).

The lipid content of immature oocytes measured soon after follicular aspiration was 9.9 ± 2.9 (expressed as the mean fluorescence intensity). After maturation, the lipid droplet content in oocytes cultured in medium containing whole estrous serum increased more than twofold compared to immature oocytes (22.8 ± 5.0; P< 0.05). In contrast, oocytes that were matured in medium containing delipidated estrous serum showed a significantly lower content of lipid droplets (7.6 ± 1.7; P< 0.05), which was similar to the levels measured in immature oocytes (P = NS). Fig 2 shows the results of lipid droplet localization and quantification of immature, control whole, and delipidated *in vitro* matured COCs after BODIPY 493/503 staining. Differences found in the oocyte neutral lipid content did not influence subsequent embryo development (Table 3). No significant differences (P = NS) were observed in the cleavage rate, developmental rates on Day 6 and Day 8, or hatching rate between groups.

**Table 3:** *In vitro* ovine embryo development after maturation of COCs supplemented with control whole or delipidated estrous sheep serum (Experiment 1, seven replicates).

	Oocytes (n)	Day 2 Cleavage rate (%)	Day 6 Morulae and blastocysts (%)	Day 8 Blastocysts (%)	Day 8 Hatching rate (%)
Control whole serum	452	69.1 ± 7.3	33.4 ± 4.9	27.0% ± 6.9	44.6 ± 9.9
Delipidated serum	414	70.8 ± 7.3	31.9 ± 4.2	29.8 ± 6.2	50.4 ± 13.2

P = NS.

### Experiment 2

**Fig 3:**
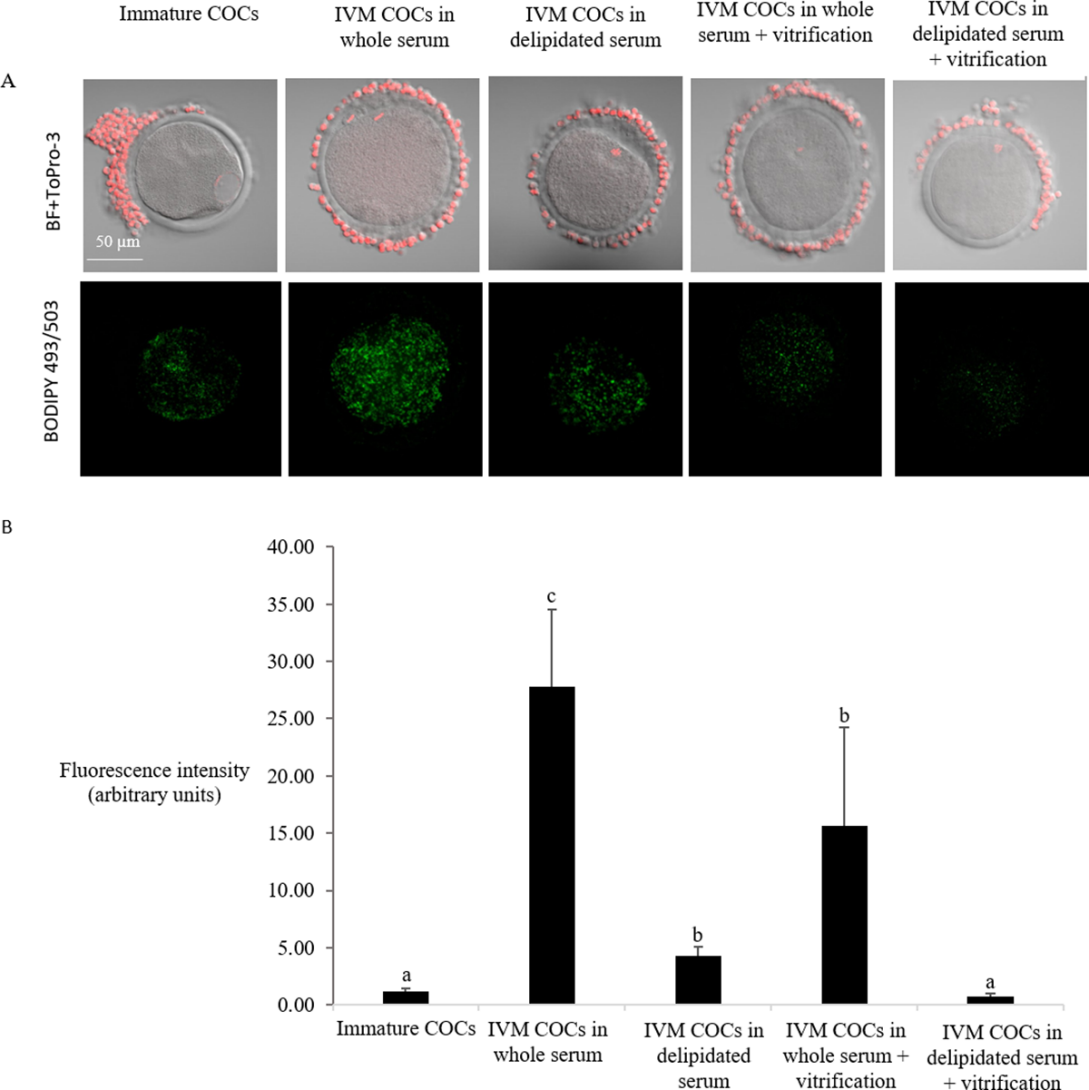
Effect of vitrification on lipid droplet content of partially denuded COCs after IVM under different lipid content conditions (Experiment 2). a) Nuclei of cells were stained with ToPro-3 (red) and merged with bright field image (BF). Neutral Lipid staining with BODIPY 493/503 (green) shows a decrease in lipid droplets of oocytes after vitrification. b) Comparison of the lipid content of immature (Immature, n = 12) *versus* IVM COCs in a medium supplemented with control whole (n = 16) or delipidated estrous sheep serum (n = 19) before or after vitrification (control whole serum+ vitrification group, n = 12; Delipidated serum+ vitrification n = 11). Values are expressed as average of BODIPY fluorescence intensity in the ooplasma per area ± SEM. Different superscripts indicate significant differences (P< 0.05).

Oocyte lipid content assessed by LD staining before and after vitrification are shown in Fig 3. Lipid droplets in oocytes exhibited reduced fluorescence after vitrification when matured *in vitro* with either medium supplemented with control whole serum or delipidated serum. Despite IVM treatment, oocytes subjected to vitrification exhibited reduced competence after *in vitro* fertilization and culture, with lower cleavage rate, embryo development rates on Day 6 and Day 8, and hatching rate when compared with the non-vitrified oocytes (P<0.05). No significant differences (P = NS) were found among non-vitrified groups, which is in agreement with the results obtained in Experiment 1 (serum effect: whole vs. delipidated). No interaction between vitrification and the type of serum (whole or delipidated) were found for cleavage, embryo development, and hatching rates (P = NS) (Table 4).

**Table 4:** *In vitro* embryo development of ovine oocytes subjected to IVM under different lipid content conditions induced by supplementation of whole or delipidated serum followed by vitrification (Experiment 2).

		Oocytes (n)	Day 2 Cleavage rate (%)	Day 6 Morulae and blastocysts (%)	Day 8 Blastocysts (%)	Day 8 Hatching rate (%)
**Main effect: Lipid content**						
	Control whole serum	632	53.3 ± 6.3^a^	34.7 ±4.6^a^	22.4 ±4.5^a^	24.6 ±6 ^a^
	Delipidated serum	648	54.6.2 ± 6.2^a^	34.6 ±4.4^a^	21.9 ±4.3^a^	29.2 ±6.5^a^
**Main effect: vitrification**						
	No vitrification	579	80.3 ± 2.9^a^	53.8 ±2.4^a^	41.71% ±2.6^a^	48.68 ± 4.9^a^
	Vitrification	701	27.2 ± 3.6^b^	15.5 ±2.4^b^	2.7% ±0.9^b^	5.1 ± 4.0^b^
Vitrification*serum interaction		NS	NS	NS	NS	NS

For the same column within main or simple effects, a vs. b differ (P < 0.05)

### Experiment 3

**Fig 4:**
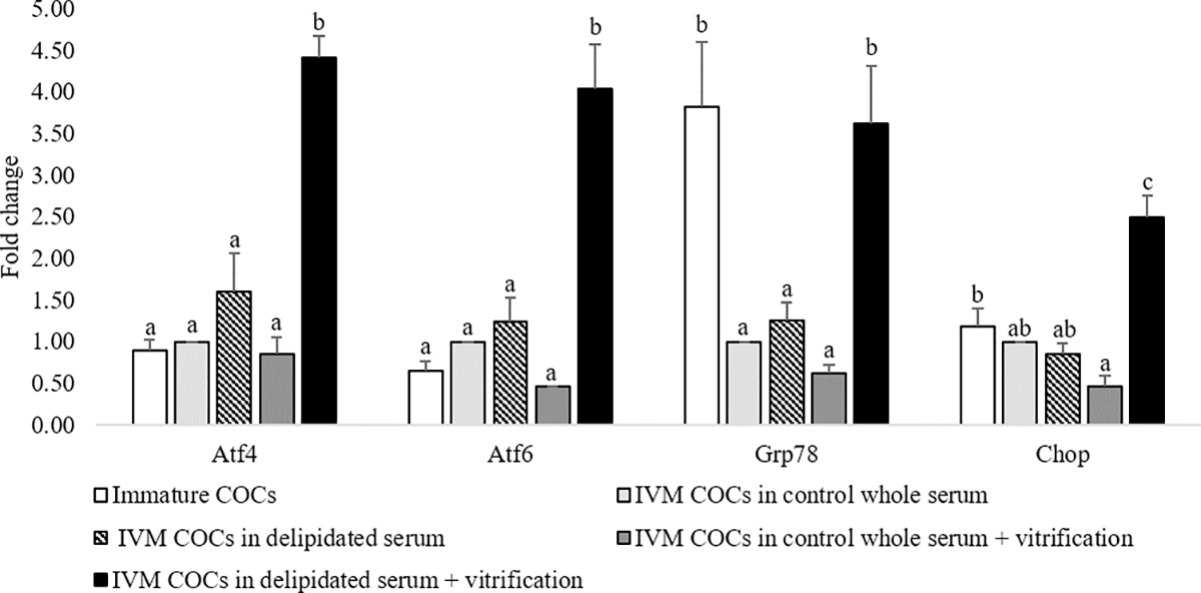
Expression of endoplasmic reticulum (ER) stress genes induced in cumulus oocytes complexes (COCs) submitted to *in vitro* maturation (IVM) in whole or delipidated serum with subsequent vitrification. Total RNA was extracted from denuded COCs, and expression of ER stress marker genes (*ATF4*, *GRP7878*, *ATF6* and *CHOP10*) was determined by qPCR. Gene expression of 5 experimental groups, interaction was found when COCs were matured in delipidated serum and subsequently vitrified. Within the same gene, different letters indicates significant differences (P< 0.05). Mean ± SEM is expressed as fold change compared with calibrator sample (control whole serum).

The expression of ER stress genes was affected by the serum type used during IVM of COCs as well as by the vitrification/warming process, with interaction between both main effects (P<0.05). The expression of classic ER stress markers (*ATF4*, *ATF6*, *GRP78*, and *CHOP10*) examined in COCs exposed to IVM media supplemented with control whole or delipidated serum and either submitted to vitrification or not is shown in Fig 4. The COCs matured in delipidated serum and subjected to vitrification exhibited increased expression of *ATF4* (4.4 fold), *ATF6* (4.0 fold), *GRP78* (3.6 fold), and *CHOP10* (2.5 fold) compared with COCs matured in IVM media supplemented with control whole serum (calibrator sample). No significant differences (P = NS) were found in the expression levels of those ER stress markers in COCs matured in control whole serum and subject to vitrification (control whole serum + vitrification group). COCs matured in delipidated serum (non-vitrified group) exhibited similar expression levels of the four ER stress markers respect to the control whole serum group. These results demonstrate that vitrification induces ER stress in COCs when they are previously matured *in vitro* in medium deprived of lipids.

## Discussion

The current study demonstrates that COCs matured *in vitro* in medium supplemented with delipidated estrous sheep serum contain fewer cytoplasmic lipid droplets than those matured in medium supplemented with whole estrous serum. However, reduced numbers of cytoplasmic lipid droplets did not improve oocyte competence and embryo production in fresh and vitrified oocytes. While the vitrification process impairs oocyte developmental competence, the serum type (delipidated or whole) used during IVM did not appear to have any adverse effects. Notably, mRNA expression levels of ER stress genes increased significantly after vitrification of oocytes but only when maturation was performed with medium supplemented with delipidated serum.

We found that the standard method used for *in vitro* maturation of COCs with a medium supplemented with whole estrous sheep serum increased oocyte neutral lipid content in comparison with immature oocytes. This increase was prevented by serum delipidation, since oocytes subjected to IVM medium with partially delipidated serum had less lipid droplets than COCs matured in control whole serum. Lipid droplets are intracellular sites of neutral lipid storage, which have been shown to play an important role in the metabolism of lipids and cellular energy homeostasis [36]. During *in vitro* maturation, serum lipids are incorporated into the oocyte cytoplasm [37], with the presence of lipids in culture media causing an increase in the number of lipid droplets in the produced embryos [38]. Although lipid droplets play a pivotal role during oocyte maturation, since oxidative phosphorylation is the main pathway to produce ATP [7], high accumulation of lipid droplets content has been correlated with poorer cryosurvival rates and reduced development competence of oocytes.[39]. In this study, we demonstrate that *in vitro* maturation of COCs in medium containing whole estrous serum increase lipid accumulation in matured oocytes, a finding that could be important for oocyte cryotolerance and embryo development.

Studies show that high lipid content in IVM oocytes correlates with reduced cryotolerance, therefore different strategies have been used to reduce numbers of lipid droplets in oocytes. One way to regulate the lipid content is the use of serum free media, which contains restricted nutrients, during IVM [1]. However, efforts to reduce lipid content in the oocytes and embryos, have been met with limited success and resulted in lower oocyte competence [40], [41]. Unlike other approaches, the protocol described herein for delipidation of estrous sheep serum was effective in decreasing levels of Triglycerides, total Cholesterol, and NEFAs. To our knowledge this is the first study to use the Cleanascite HC method to generate estrous sheep serum yielding significantly reduced lipid levels. Subsequent use of the partially delipidated serum as supplemented in IVM media resulted in effective reduction of oocyte lipid content. The advantage of this method over other traditional methods (i.e. chloroform) includes increased feasibility and reduced toxicity and biosafety concerns [42]. Similar results have been found when oocyte delipidation is achieved by stimulating lipid metabolism (i.e. L-Carnitine) [43]. The approach of encouraging embryos and oocytes to deplete intracellular lipids to increase cryosurvival rates has been shown to be a more benign alternative to mechanical delipidation and safer for oocytes and embryos [1]. Furthermore, our protocol enables simultaneous delipidation of large numbers of oocytes and does not require micromanipulation, thereby increasing efficiency and improving viability of oocytes.

The current study shows that oocyte competence was affected by cryopreservation independently of the lipid environment in which the COCs were matured. Cryopreservation protocols are not well established for oocytes, especially in domestic animals due to their high lipid content.[44]. Ultra-rapid vitrification methods have made it possible to overcome some challenges associated with oocyte cryopreservation. Specifically, the use of Cryotop with minimum volume of vitrification (<0.1 μl) and high cooling rates (22.800°C min^-1^) has allowed human oocytes to obtain post-warming survival rates of >90% and blastocyst rates of roughly 50% [29]. Compared to human oocytes, oocytes from some species of domestic animals have high lipid content which increases their sensitivity to cooling processes and exacerbates outcomes after IVM [1]. Overall, species that have higher lipid content in their ooplasma have lower survival rates following cryopreservation [39]. As expected, we found that vitrified oocytes that had been in IVM medium containing whole serum showed reduced cleavage rates and embryo development compared to fresh control oocytes that were not subjected to vitrification. Interestingly, although incorporation of partially delipidated serum in the IVM medium resulted in lower lipid content in the matured oocytes, it did not improve outcomes after vitrification including cleavage rates and embryo development rates in comparison to IVM oocytes with control whole serum medium. Collectively, our results show that reducing lipid content during the COC maturation process prior to cryopreservation is not sufficient to improve oocyte competences following, suggesting that lipid content is one factor amongst many variables/factors that can lead to reduced tolerance to cryopreservation. Some studies attribute the lower cryotolerance of *in vitro* produced embryos to an imbalance of oxidation-reduction metabolism leading to greater accumulation of reactive oxygen species in the culture medium and reduced survival rates of embryos [45]. Our current results suggest that a negative correlation exists between lipid content and oocyte cryotolerance. It is well known that embryos derived from live animals have fewer lipid droplets compared to IVP embryos [46]. This difference in lipid neutral content may explain, in part, the differences in cryotolerance between *in vivo* versus *in vitro* embryos [1]. Some studies have reported similar differences in bovine and porcine oocytes matured *in vivo* vs. *in vitro* [47,48]. These observations support the idea that if IVM oocytes in delipidated systems could resemble *in vivo* matured oocytes in terms of lipid droplets content and that cryotolerance could be improved. However, despite the lower lipid droplet content found in those COCs subjected to IVM with partially delipidated serum, no differences in oocyte competence were found. It is likely that either the variations observed are insufficient to alter oocytes’ cryotolerance to vitrification, or that a reduction of lipid droplets using our experimental conditions is not a key factor to overcome damages associated with vitrification.

Finally, we demonstrate that vitrification induces greater expression of ER stress genes in COCs matured in a medium with reduced lipid content. The ER folding capacity can be disturbed by biological stimuli, resulting in an accumulation of misfolded and unfolded proteins in the ER lumen and ER stress [32]. ER stress triggers a homeostasis response, referred to as the unfolded protein response (UPR) [49], which involves the activation of ER transmembrane signaling molecules (PERK, IRE1 and ATF6). Activation of these three master regulators of UPR influence the transcription of several genes involved in UPR. IRE1α regulates the splicing of XBP1 promoting components of ER-associated protein degradation (ERAD) as Bip/GRP78. Activation of PERK favor translation of ATF4, which regulates genes involved in protein folding, degradation, and apoptosis, including CHOP. In this study, we have selected downstream regulators *ATF4*, *CHOP*, and *GRP78* as representative genes of the PERK/IRE1 pathway to determine UPR activation [32]. Differences in the expression levels of UPR genes has been related with lipid droplet content in mice and expression of *GRP78* is greater when oocytes are matured in vivo [50]. Lipid peroxidation has also been linked to induction of UPR in endothelial cells [51] and augmentation in the expression levels of *XBP1* has been reported in vitrification-warmed mouse oocytes [12]. Our data revealed that *GRP78* is expressed highly in immature sheep oocytes compared to COCs that were matured *in vitro*. No significant differences were found in the expression levels of *ATF4*, *ATF6*, *GRP78*, and *CHOP10* in COCs that were matured *in vitro*—either in whole serum or in delipidated serum. Attenuation in the expression levels of *GRP78* in *in vitro* matured COCs could be a consequence of FSH supplementation in the IVM media as reported previously for mice [52]. COCs that were matured *in vitro* in IVM media supplemented with delipidated serum and vitrification upregulated the expression of *ATF4*, *ATF6*, *CHOP10*, and GRP78. It has been previously suggested that lipid droplets may serve as a site of storage to sequester unfolded or excessive proteins, thereby alleviating ER stress [53]. Therefore, the amount of lipid droplets in COCs matured in whole serum and vitrified reduced their sensitivity to ER stress induction post-warming. Another possible explanation for our results, is that oocyte competence is regulated by adaptive machineries governed by ER stress. Various genes associated with ER stress were found in oocytes and preimplantation embryos of mice and pigs, as a normal part of preimplantation embryos adaptive machineries [54,55]. However, some studies oppose the notion that the UPR response promotes oocyte competence. The inhibition of the UPR response by different inhibitors (TUDCA or Salubrinal) was shown to enhance maturation of pig and mouse oocytes by preventing ER stress mediated apoptosis in vitro [24,55], suggesting that ER stress may negatively impact oocyte developmental competence. To our knowledge, this is the first time that expression of the *ATF4*, *ATF6*, *CHOP10*, and *GRP78* genes has been evaluated in ovine oocytes. We demonstrate that vitrification can cause activation of UPR when oocytes are diminished in lipid droplets content.

In conclusion, this study demonstrates that partial delipidation of estrous sheep serum used for supplementation of IVM medium reduces the neutral lipid content and the presence of cytoplasmic lipid droplet in oocytes. However, the culture of oocytes in delipidated serum did not result in improved cryotolerance when *in vitro* matured oocytes were subjected to vitrification. COCs with reduced amounts of lipid droplets subjected to vitrification were observed to have higher expression of UPR genes. Overall, this study provides a feasible method to reduce lipid droplets in oocytes that are *in vitro* matured, and suggests the need of a revision of the idea that oocyte cryotolerance may be improved by lipid content depletion.

## References

[1] Wirtu G, Mcgill J, Crawford L, Reddy G, Bergen WG, Simon L. Targeting Lipid Metabolism to Improve Oocyte Cryopreservation (OCP) in Domestic Animals. 2013;1:15–20.

[2] Vajta G. Vitrification of the oocytes and embryos of domestic animals. Anim. Reprod. Sci. 2000;60–61:357–64. 10.1016/s0378-4320(00)00097-x 10844207

[3] Menchaca A, Barrera N, Santos PC dos, Cuadro F, Crispo M. Advances and limitations of in vitro embryo production in sheep and goats. Anim. Reprod. [Internet]. 2016;13:273–8. Available from: http://www.cbra.org.br/pages/publicacoes/animalreproduction/issues/download/v13/v13n3/p273-278(AR871).pdf

[4] Lonergan P, Fair T. Maturation of Oocytes in Vitro. Annu. Rev. Anim. Biosci. United States; 2016;4:255–68. 10.1146/annurev-animal-022114-110822 26566159

[5] Wrenzycki C, Stinshoff H. Maturation environment and impact on subsequent developmental competence of bovine oocytes. Reprod. Domest. Anim. Germany; 2013;48 Suppl 1:38–43. 10.1111/rda.12204 23962213

[6] Collado-Fernandez E, Picton HM, Dumollard Ré. Metabolism throughout follicle and oocyte development in mammals. Int. J. Dev. Biol. 2012;56:799–808. 10.1387/ijdb.120140ec 23417402

[7] Sturmey RG, Leese HJ. Energy metabolism in pig oocytes and early embryos. Reproduction. 2003;126:197–204. 12887276 10.1530/rep.0.1260197

[8] Dunning KR, Russell DL, Robker RL. Lipids and oocyte developmental competence: The role of fatty acids andβ-oxidation. Reproduction. 2014;148. 10.1530/REP-13-0251 24760880

[9] Listenberger LL, Brown D a. Fluorescent detection of lipid droplets and associated proteins. Curr. Protoc. Cell Biol. 2007;Chapter 24:Unit 24.2. 10.1002/0471143030.cb2402s35 18228510

[10] Abe H, Yamashita S, Satoh T, Hoshi H. Accumulation of cytoplasmic lipid droplets in bovine embryos and cryotolerance of embryos developed in different culture systems using serum-free or serum-containing media. Mol. Reprod. Dev. [Internet]. John Wiley & Sons, Inc.; 2002;61:57–66. Available from: 10.1002/mrd.1131 11774376

[11] Ferguson EM, Leese HJ. Triglyceride content of bovine oocytes and early embryos. J. Reprod. Fertil. 1999;116:373–8. 10615263 10.1530/jrf.0.1160373

[12] Zhao N, Liu XJ, Li JT, Zhang L, Fu Y, Zhang YJ, et al Endoplasmic reticulum stress inhibition is a valid therapeutic strategy in vitrifying oocytes. Cryobiology [Internet]. Elsevier Inc.; 2015;70:48–52. Available from: 10.1016/j.cryobiol.2014.12.001 25499542

[13] Zhou G Bin, Li N. Cryopreservation of porcine oocytes: Recent advances. Mol. Hum. Reprod. 2009 p. 279–85. 10.1093/molehr/gap016 19251762

[14] dos Santos Neto PC, Vilariño M, Barrera N, Cuadro F, Crispo M, Menchaca A. Cryotolerance of Day 2 or Day 6 in vitro produced ovine embryos after vitrification by Cryotop or Spatula methods. Cryobiology. 2015; 10.1016/j.cryobiol.2014.11.001 25448379

[15] dos Santos-Neto PC, Cuadro F, Barrera N, Crispo M, Menchaca A. Embryo survival and birth rate after minimum volume vitrification or slow freezing of in vivo and in vitro produced ovine embryos. Cryobiology. 2017;78:8–14. 10.1016/j.cryobiol.2017.08.002 28803846

[16] Shirazi A, Naderi MM, Hassanpour H, Heidari M, Borjian S, Sarvari A, et al The effect of ovine oocyte vitrification on expression of subset of genes involved in epigenetic modifications during oocyte maturation and early embryo development. Theriogenology [Internet]. Elsevier Inc; 2016;86:2136–46. Available from: 10.1016/j.theriogenology.2016.07.005 27501872

[17] Bhat MH, Yaqoob SH, Khan FA, Waheed SM, Sharma V, Vajta G, et al Open pulled straw vitrification of in vitro matured sheep oocytes using different cryoprotectants. Small Rumin. Res. 2013;112:136–40.

[18] Bhat MH, Sharma V, Khan FA, Naykoo NA, Yaqoob SH, Vajta G, et al Open pulled straw vitrification and slow freezing of sheep IVF embryos using different cryoprotectants. Reprod. Fertil. Dev. 2015;27:1175–80. 10.1071/RD14024 24871337

[19] Mullen SF, Fahy GM. A chronologic review of mature oocyte vitrification research in cattle, pigs, and sheep. Theriogenology. 2012 p. 1709–19. 10.1016/j.theriogenology.2012.06.008 22968034

[20] Hwang IS, Hochi S. Recent progress in cryopreservation of bovine oocytes. Biomed Res. Int. 2014;2014. 10.1155/2014/570647 PMC3971499 24738063

[21] Shabankareh HK, Zandi M. Developmental potential of sheep oocytes cultured in different maturation media: effects of epidermal growth factor, insulin-like growth factor I, and cysteamine. Fertil. Steril. [Internet]. Elsevier Ltd; 2010;94:335–40. Available from: 10.1016/j.fertnstert.2009.01.160 19324348

[22] Du Y, Pribenszky CS, Molnár M, Zhang X, Yang H, Kuwayama M, et al High hydrostatic pressure: A new way to improve in vitro developmental competence of porcine matured oocytes after vitrification. Reproduction. 2008;135:13–7. 10.1530/REP-07-0362 18159079

[23] Pribenszky C, Lin L, Du Y, Losonczi E, Dinnyes A, Vajta G. Controlled stress improves oocyte performance—cell preconditioning in assisted reproduction. Reprod. Domest. Anim. Germany; 2012;47 Suppl 4:197–206. 10.1111/j.1439-0531.2012.02076.x 22827371

[24] Wu LL, Russell DL, Norman RJ, Robker RL. Endoplasmic reticulum (ER) stress in cumulus-oocyte complexes impairs pentraxin-3 secretion, mitochondrial membrane potential (DeltaPsi m), and embryo development. Mol. Endocrinol. [Internet]. 2012;26:562–73. Available from: http://www.ncbi.nlm.nih.gov/pubmed/22383462 22383462 10.1210/me.2011-1362 PMC5417137

[25] Gordon I. Laboratory production of cattle embryos 2nd edition CAB Int. Univ. Press 2003.

[26] Leroy J, Genicot G, Donnay I, Van Soom A. Evaluation of the lipid content in bovine oocytes and embryos with nile red: a practical approach. Reprod.Domest.Anim. 2005;40:76–8. 10.1111/j.1439-0531.2004.00556.x 15655006

[27] Menchaca A, Rubianes E. New treatments associated with timed artificial insemination in small ruminants. Reprod. Fertil. Dev. 2004;16:403–13. 10.10371/RD04037 15315739

[28] Parrish JJ, Susko-Parrish JL, Leibfried-Rutledge ML, Critser ES, Eyestone WH, First NL. Bovine in vitro fertilization with frozen-thawed semen. Theriogenology. United States; 1986;25:591–600. 16726150 10.1016/0093-691x(86)90143-3

[29] Kuwayama M, Kato O, Leibo SP, Genetics P. Article Highly effi cient vitrifi cation method for cryopreservation of human oocytes. Biomedicine. 2005;11:300–8. 10.1016/s1472-6483(10)60837-1 16176668

[30] Tamura AN, Huang TTF, Marikawa Y. Impact of vitrification on the meiotic spindle and components of the microtubule-organizing center in mouse mature oocytes. Biol. Reprod. [Internet]. 2013;89:112 Available from: http://www.ncbi.nlm.nih.gov/pubmed/24025740 24025740 10.1095/biolreprod.113.108167 PMC4076379

[31] Aardema H, Vos PL a M, Lolicato F, Roelen B a J, Knijn HM, Vaandrager AB, et al Oleic acid prevents detrimental effects of saturated fatty acids on bovine oocyte developmental competence. Biol. Reprod. [Internet]. 2011;85:62–9. Available from: http://www.ncbi.nlm.nih.gov/pubmed/21311036 21311036 10.1095/biolreprod.110.088815

[32] Oslowski CM, Urano F. Measuring ER stress and the unfolded protein response using mammalian tissue culture system. Methods Enzymol. [Internet]. 2013;490:71–92. Available from: http://www.sciencedirect.com/science/article/pii/B9780123851147000040 10.1016/B978-0-12-385114-7.00004-0 PMC3701721 21266244

[33] O’Connor T, Wilmut I, Taylor J. Quantitative evaluation of reference genes for real-time pcr during in vitro maturation of ovine oocytes. Reprod. Domest. Anim. 2013;48:477–83. 10.1111/rda.12112 23066791

[34] Rutledge RG, Côté C. Mathematics of quantitative kinetic PCR and the application of standard curves. Nucleic Acids Res. 2003;31:e93 12907745 10.1093/nar/gng093 PMC169985

[35] Pfaffl MW. A new mathematical model for relative quantification in real-time RT-PCR. Nucleic Acids Res. [Internet]. 2001;29:e45 Available from: http://www.ncbi.nlm.nih.gov/pubmed/11328886 11328886 10.1093/nar/29.9.e45 PMC55695

[36] Welte MA. Expanding roles for lipid droplets. Curr. Biol. [Internet]. Elsevier Ltd; 2015;25:R470–81. Available from: 10.1016/j.cub.2015.04.004 26035793 PMC4452895

[37] Kim JY, Kinoshita M, Ohnishi M, Fukui Y. Lipid and fatty acid analysis of fresh and frozen-thawed immature and in vitro matured bovine oocytes. Reproduction. 2001;122:131–8. 11425337

[38] Leroy J, Genicot G, Donnay I, Soom A Van. Short Communication Evaluation of the Lipid Content in Bovine Oocytes and Embryos with Nile Red: a Practical Approach. 2005;78:76–8. 10.1111/j.1439-0531.2004.00556.x 15655006

[39] Prates EG, Nunes JT, Pereira RM. A role of lipid metabolism during cumulus-oocyte complex maturation: Impact of lipid modulators to improve embryo production. Mediators Inflamm. Hindawi Publishing Corporation; 2014;2014. 10.1155/2014/692067 PMC3964899 24733963

[40] Accorsi MF, Leão BC da S, Rocha-Frigoni NA de S, Perri SHV, Mingoti GZ. Reduction in cytoplasmic lipid content in bovine embryos cultured in vitro with linoleic acid in semi-defined medium is correlated with increases in cryotolerance. Zygote. 2015;24:485–94. 10.1017/S0967199415000428 26350684

[41] Mishra A, Gupta PSP, Sejian V, Reddy IJ, Ravindra JP. Maturation timing and fetal bovine serum concentration for developmental potential of sheep oocytes in vitro. 2016;54:630–3. 30084562

[42] Castro AR, Morrill WE, Pope V. Lipid removal from human serum samples. Clin. Diagn. Lab. Immunol. 2000;7:197–9. 10702492 10.1128/cdli.7.2.197-199.2000 PMC95848

[43] Dunning KR, Robker RL. Promoting lipid utilization with l-carnitine to improve oocyte quality. Anim. Reprod. Sci. [Internet]. Elsevier B.V.; 2012;134:69–75. Available from: 10.1016/j.anireprosci.2012.08.013 22917873

[44] Woods EJ, Benson JD, Agca Y, Critser JK. Fundamental cryobiology of reproductive cells and tissues. Cryobiology. 2004;48:146–56. 10.1016/j.cryobiol.2004.03.002 15094091

[45] Sudano MJ, Paschoal DM, da Silva Rascado T, Magalhães LCO, Crocomo LF, de Lima-Neto JF, et al Lipid content and apoptosis of in vitro-produced bovine embryos as determinants of susceptibility to vitrification. Theriogenology [Internet]. Elsevier Inc.; 2011;75:1211–20. Available from: 10.1016/j.theriogenology.2010.11.033 21247620

[46] Seidel GE. Modifying oocytes and embryos to improve their cryopreservation. Theriogenology. 2006 p. 228–35. 10.1016/j.theriogenology.2005.09.025 16263160

[47] Del Collado M, Saraiva NZ, Lopes FL, Gaspar RC, Padilha LC, Costa RR, et al Influence of bovine serum albumin and fetal bovine serum supplementation during in vitro maturation on lipid and mitochondrial behaviour in oocytes and lipid accumulation in bovine embryos. Reprod. Fertil. Dev. 2016;28:1721–32. 10.1071/RD15067 25986410

[48] Kikuchi K, Ekwall H, Tienthai P, Kawai Y, Noguchi J, Kaneko H, et al Morphological features of lipid droplet transition during porcine oocyte fertilisation and early embryonic development to blastocyst in vivo and in vitro. Zygote. 2002;10:355–66. 12463532 10.1017/s0967199402004100

[49] Volmer R, Ron D. Lipid-dependent regulation of the unfolded protein response. Curr. Opin. Cell Biol. [Internet]. Elsevier Ltd; 2015;33:67–73. Available from: 10.1016/j.ceb.2014.12.002 25543896 PMC4376399

[50] Yang X, Wu LL, Chura LR, Liang X, Lane M, Norman RJ, et al Exposure to lipid-rich follicular fluid is associated with endoplasmic reticulum stress and impaired oocyte maturation in cumulus-oocyte complexes. Fertil. Steril. [Internet]. Elsevier Inc.; 2012;97:1438–43. Available from: 10.1016/j.fertnstert.2012.02.034 22440252

[51] Vladykovskaya E, Sithu SD, Haberzettl P, Wickramasinghe NS, Merchant ML, Hill BG, et al Lipid peroxidation product 4-hydroxy-trans-2-nonenal causes endothelial activation by inducing endoplasmic reticulum stress. J. Biol. Chem. 2012;287:11398–409. 10.1074/jbc.M111.320416 22228760 PMC3322871

[52] Babayev E, Lalioti M D, Favero F, Seli E. Cross-talk between FSH and endoplasmic reticulum stress: A mutually suppressive relationship. Reprod. Sci. 2016;23:352–64. 10.1177/1933719115602770 26342052 PMC5933091

[53] Zhang X, Zhang K. Endoplasmic reticulum stress-associated lipid droplet formation and type II diabetes. Biochem. Res. Int. 2012;2012. 10.1155/2012/247275 PMC3299243 22506114

[54] Michalak M, Gye MC. Endoplasmic reticulum stress in periimplantation embryos. Clin. Exp. Reprod. Med. 2015;42:1–7. 10.5653/cerm.2015.42.1.1 25874167 PMC4390675

[55] Zhang JY, Diao YF, Oqani RK, Han RX, Jin DI. Effect of Endoplasmic Reticulum Stress on Porcine Oocyte Maturation and Parthenogenetic Embryonic Development In Vitro. Biol. Reprod. 2012;86:128–128. 10.1095/biolreprod.111.095059 22190710

